# Novel Peptide NIRF Optical Surgical Navigation Agents for HNSCC

**DOI:** 10.3390/molecules24173070

**Published:** 2019-08-23

**Authors:** Haiming Ding, Shankaran Kothandaraman, Li Gong, Chadwick L. Wright, Quintin Pan, Theodore Teknos, Michael F. Tweedle

**Affiliations:** 1 Department of Radiology, The Wright Center of Innovation in Biomedical Imaging, The Ohio State University, Columbus, OH 43210, USA; 2 Seidman Cancer Center, University Hospitals, Cleveland, OH 44106, USA

**Keywords:** HNSCC, optical imaging, HN1, peptide

## Abstract

Head and neck squamous cell carcinoma (HNSCC) survival rates have not improved in a decade, with a 63% 5-year recurrence rate after surgery, making HNSCC a compelling indication for optical surgical navigation (OSN). A promising peptide, HN1, targeted and internalized in human HNSCC cells in multiple laboratories, but was slow (24 h) to accumulate. We modified HN1 and explored structural variables to improve the uptake kinetics and create IRdye800 adducts useful for OSN. Eleven new molecules were synthesized and characterized chemically, in human HNSCC cells (Cal 27), and in HNSCC xenograft mice. Cal 27 flank xenografts in Balb/c nude mice were imaged for 3–48 h after 40 nmol intravenous doses of IR800-labeled molecules. Cell uptake kinetics in the 1–2 h window incubated at 1–10 μM were independent of the dye label (FITC, Cy5, or IR800), but increased markedly with additional N-terminal lipophilic substitution, and after resequencing the peptide to separate polar amino acids and move the lysine-dye more centrally. Microscopy confirmed the strong Cal 27 cell binding and demonstrated primarily cytosolic and membrane localization of the fastest peptide, 4Iphf-HN17. 4Iph-HN17-IR800 showed 26-fold greater rate of uptake in cells than HN1-IR800, and far stronger OSN imaging intensity and tumor to background contrast in mice, suggesting that the new peptide is a promising candidate for OSN of HNSCC.

## 1. Introduction

Head and neck squamous cell carcinoma (HNSCC) is the sixth most common malignancy in the United States. Despite advances in diagnosis and therapy, there has been no significant improvement in its 5-year relative survival rate for more than a decade [1]. Surgery is the primary treatment modality for HNSCC [2]. Successful surgery (1–10 mm of clear margins at pathology) still results in 50%–60% chance of local regional recurrence within 5 years [3,4], and recurrence can be as high as 90% if the margin is positive [4], which it is in ~25% of patients [5]. While pre-surgical radiological imaging and experience aid the surgical planning, intraoperative margin decisions are made primarily by vision and palpation, with disfigurement as a primary side effect of surgery. Thus, any practical method that improves the accuracy of intraoperative detection of tumor tissue along the margin is likely to reduce recurrence risk, increase survival, and minimize disfigurement caused by excessive normal tissue removal.

Recently, Optical Surgical Navigation (OSN) with Near Infrared Flourescence (NIRF) imaging has emerged to fill the need for real time intraoperative imaging. This technique is sensitive enough to detect nM dicyanine dyes targeted to cancer cells. Two IRdye800-labeled antibodies are currently undergoing clinical investigation in HNSCC: anti-EGFR and anti-CD147 [6,7,8,9,10]. Antibodies with nM affinity for target proteins are relatively simple to discover, but have recognized deficiencies related to their large size, which makes them slow to clear background tissue [11], and in non-nuclear imaging applications like OSN the large mass dose makes them expensive to commercialize.

Small peptides can have equally strong affinity and specificity for targets, faster pharmacokinetics, and are less expensive than antibodies to scale up and develop [12,13,14]. Peptides based upon cyclic arginine-glycine-aspartate (RGD) have been identified that target integrins overexpressed on the surface of human HNSCC cells. Hsiao et al. discovered an αvβ6 specific peptide via biopanning phage [15]. An HNSCC-binding peptide (HBP-1), discovered by Nothelfer et al., is composed of RGD and LXXL motifs, and also binds ανβ6 [16]. Atallah et al. reported that a Cy5-labeled RGD-based peptide could detect tiny tumor tissues unrecognized by human eyes during resection in a mouse cheek orthotopic model, and that removal of those tissues lengthened mice survival [17].

The oldest peptide developed for HNSCC, HN1, is the only one whose HNSCC cell affinity has been reproduced in multiple laboratories. HN1 was discovered through phage-display screening of human HNSCC cancer cells against normal human fibroblasts, and validated in human HNSCC cells in vitro, ex vivo human cancer tissue, and in vivo mouse tumor xenografts, using an optically labeled analog, HN1-FITC [18]. HN1 was shown to internalize in HNSCC cells in μM concentrations and conjugation of HN1 with drugs enhanced the drugs’ therapeutic effects [19,20]. However, HN1 was very slow to internalize requiring >24 h in cell culture [21]. The specific mechanism of action of HN1 is uncertain, but it behaves like a cell penetrating peptide (CPP), internalizing in cells at μM concentration even when covalently conjugated with dyes or drugs, and it has been categorized in a CPP review [22] and listed in a large validated CPP database [23]. We report herein, systematic studies to create new HN1-related peptide molecules that have a greatly increased uptake and internalization rate within HNSCC cells compared to HN1. We also use, unlike prior studies, a clinically validated NIRF dye as the fluorescent label, IRdye800 (IR800) [8] in place of FITC and Cy5, which emit at wavelengths too low to be detected in clinical imagers. The best new molecule exhibits the tumor-targeting features of HN1 in vitro and in vivo but with 26-fold internalization rate increase at 1 h in cultured cells, and much stronger emission intensity in mice xenograft HNSCC tumor imaging.

## 2. Results

### 2.1. Syntheses and Nomenclature

**Figure 1:**
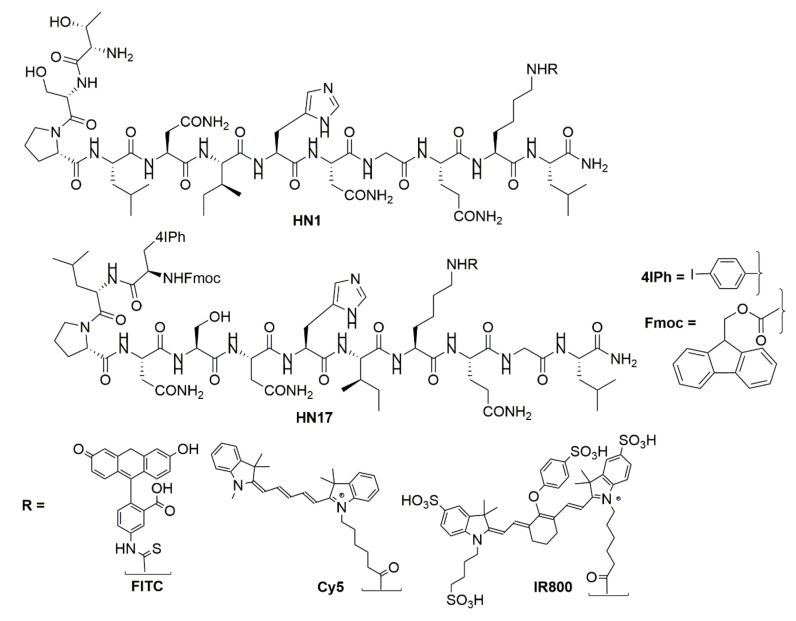
Chemical structures of HN1, 4Iphf-HN17, and the dye labels, FITC, Cy5, and IR800.

Table 1 contains the peptides synthesized and studied along with their name abbreviations, and Figure 1 shows the chemical structures of the HN1 and 4Iphf-HN17 peptides and of the lysine-conjugated dyes. Three groups of molecules were studied: the original HN1 sequence, a second sequence based on the original negative control “jumbled” peptide, HNJ, wherein the seven polar amino acids are clustered adjacent at the N-terminus, and a new group made with a novel sequence of the same amino acids, HN17. We retained the original names of the HN1 and HNJ derived peptides, adding the conjugated fluorescent dye as a suffix, e.g., HN1-FITC, and abbreviated additional moieties at the N-terminus as: f for Fmoc and 4Iph for 4-iodophenyl, e.g., f-HN1-FITC. All dye conjugations were to the lysine.

### 2.2. In Vitro Studies

**Figure 2:**
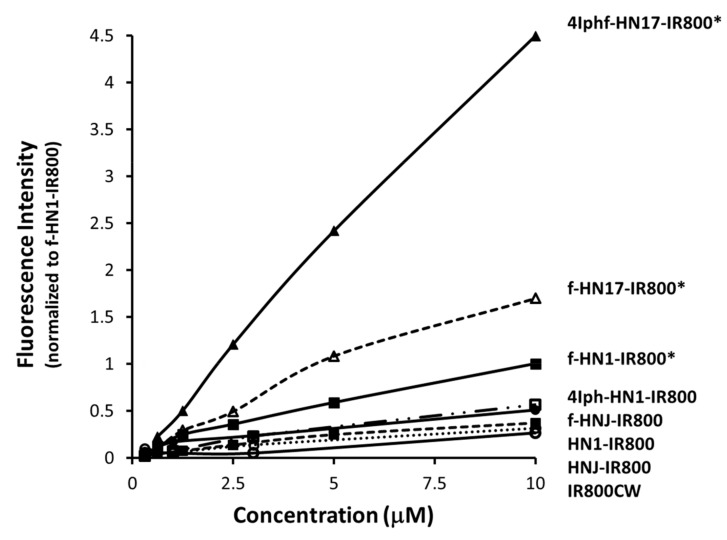
Relative uptake of peptides as IR800 conjugates, as a function of incubation concentration in cultured Cal 27 cells measured by fluorescence of the washed, lysed cells after 2 h of incubation. Cal 27 cells were incubated with the agents at 0.625–10 μM. Cell uptake is normalized to f-HN1-IR800 at 10 μM (1.0 on the vertical scale). The bottom most curve is the unconjugated IR800 dye as the carboxylate. * indicates statistical difference from HN1-IR800 at *p* < 0.05. Tables S2 and S3 detail methodology and report numerical data points in the figure, with uncertainties and *p* values.

Screening for the rate of uptake was performed in Cal 27 cells with 1–2 h incubations in a concentration series up to 10 μM, using f-HN1-IR800 as an internal control to normalize the data. The 2 h incubations of the full series of new molecules’ results are shown in Figure 2. Similar to the findings of Hong et al. [18] for HNJ-FITC, the HNJ-IR800 showed poor uptake, no better than the free IRDye800-CW (carboxylate analog). Addition of an Fmoc to the N-terminus of the sequence, f-HNJ-IR800, did not significantly increase the uptake, but adding Fmoc to the HNSCC-specific HN1-dye significantly increased uptake. The 4Iph was then tested to add lipophilicity and polarizability using an iodine atom. The series of molecules spanned a >12-fold range in 0–10 μM uptake in 2 h incubations as shown in Figure 2, and a 26-fold range in 1 h incubations (Figure S3). The Fmoc and 4Iph N-terminal substitutions and resequencing to create HN17 independently increased the rate of Cal 27 cell uptake.

**Figure 3:**
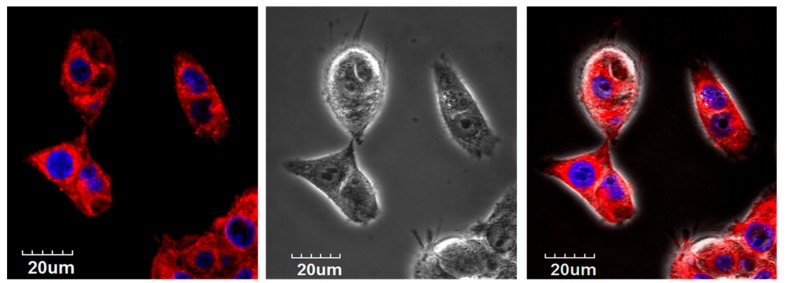
Confocal microscopy on live Cal 27 cells L to R: fluorescent, bright field, and merged images. The top row shows internalization of 4Iphf-HN17-Cy5 (red) into cytosol. Red: 4phlf-HN17-Cy5; Blue: Hoechst 33,342 stained nuclei.

Cellular uptake of 4Iphf-HN17-IR800 was confirmed by a fluorescence microscopic assay on live Cal 27 cells (Figure S4). Cal 27 cells were bright with uptake and retention at 1 and 24 h, with emission intensity brighter at 1 h than at 24 h. In contrast, there was little fluorescent signal captured in the Cal 27 cells incubated with HN1-IR800, HNJ-IR800, or IRDye800-CW at either 1 or 24 h. The live cell confocal images in Figure 3 confirmed that the uptake of 5 μM 4Iphf-HN17-Cy5 in Cal 27 cells after 1.5 h is internalized into cytoplasm. Internalized peptide appeared mostly as ≤1 μm sized punctate structures, suggesting endocytosis of the peptide.

**Figure 4:**
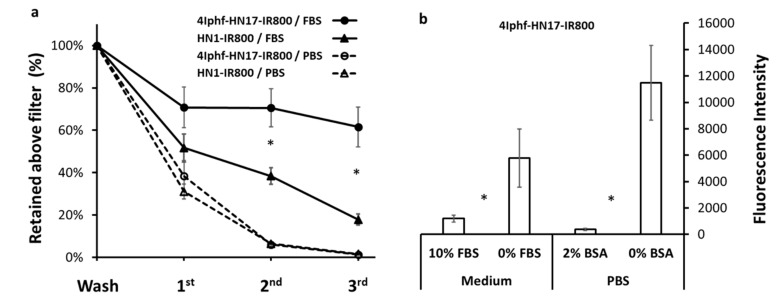
(**a**) Comparison of binding of 4Iphf-HN1-IR800 and HN1-IR800 to proteins in FBS (fetal bovine serum). The horizontal axis refers to the number of washings of the filtered fraction. Bound agents were retained above the 10 kDa molecular size filter. * 4Iphf-HN17-IR800 was retained to a significantly greater degree (*p* < 0.05) than HN1-IR800. (**b**) 4Iphf-HN17-IR800 was incubated for 1 h with Cal27 cells with and without an albumin source. Demonstration that FBS or BSA (bovine serum albumin) presence in the incubation media during incubations with Cal 27 cells diminishes the cell uptake, eliminating albumin uptake as a mechanism for peptide uptake. * *p* < 0.05.

Prior to in vivo studies, we ascertained the serum protein binding potential and serum stability of two derivatives. Figure 4a shows that 4Iphf-HN17-IR800 is a stronger protein binder than HN1-IR800. There was approximately 70% and 52% of 25 μM peptide bound to FBS (fetal bovine serum) proteins in 100% FBS, respectively (*p* < 0.05). To verify that the protein binding did not provide the primary mechanism of uptake, we measured Cal 27 cell uptake with and without FBS and BSA (bovine serum albumin) in the media (Figure 4b), finding that the FBS and BSA substantially diminished the cell uptake (*p* < 0.05), probably by reducing the concentration of peptide available for cell binding.

**Figure 5:**
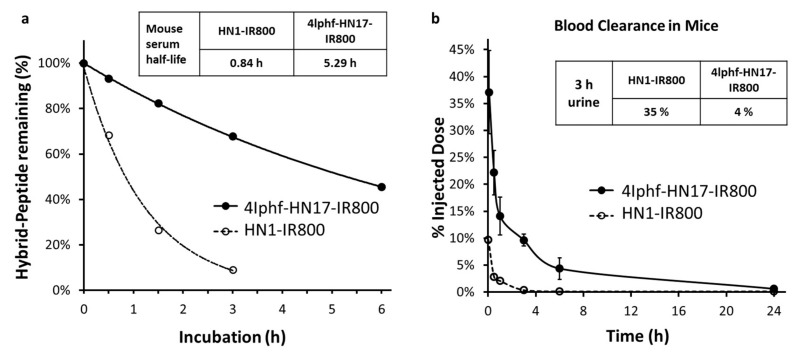
(**a**) Metabolism of 4Iphf-HN1-IR800 and HN1-IR800 in 100% mouse serum. The peptide quantities were determined by peak areas in HPLC chromatograms. (**b**) Blood clearance of peptides from mice administered 40 nmol doses intravenously, and urine accumulation total at 3 h post administration.

Serum stability was investigated as an important factor for an agent to maintain its effective concentration in vivo. As shown in Figure 5a 4Iphf-HN17-IR800 had a serum half-life 6.3-fold longer than HN1-IR800 in mouse serum at 37 °C (5.29 h vs. 0.84 h). In aqueous buffer, the peptides were stable (unchanged in HPLC) for 2 days, and also for 2 weeks frozen at −20 °C.

### 2.3. In Vivo Studies

Blood clearance data in mice for 4Iphf-HN17-IR800 and HN1-IR800 are shown in xref Figure 5b and represent the range of behavior for the series (Figure S5). Both peptides displayed a rapid initial distribution phase followed by a slower elimination phase with nearly complete blood clearance by 24 h. 4Iphf-HN17-IR800 exhibited a significantly higher plasma concentration within the first 3 h (37.1% vs. 9.7% at 5 min, 22.2% vs. 2.8% at 0.5 h, 14.1% vs. 2.1% at 1 h, and 9.6% vs. 0.3% at 3 h; *p* < 0.05 at all time points). 4Iphf-HN17-IR800 had only 4.4% ID in the urine of the mice within the first 3 h after intravenous administration compared to 35% for HN1-IR800.

**Figure 6:**
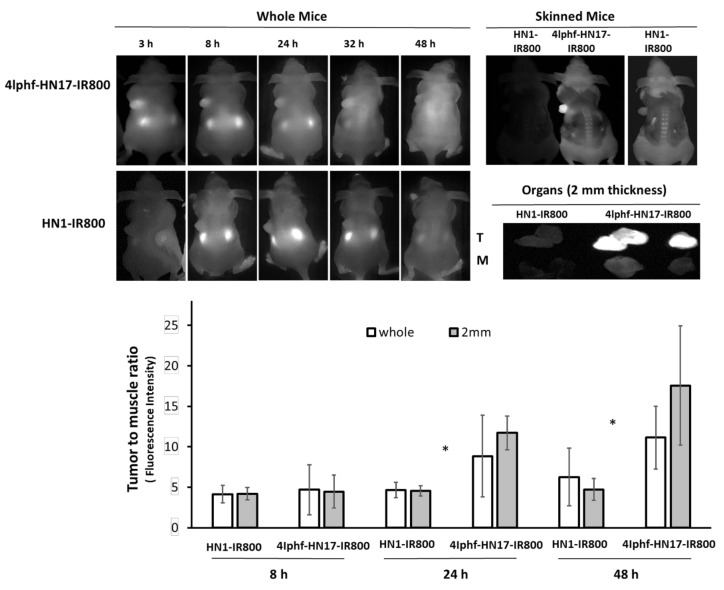
Fluobeam optical images of live whole mice bearing Cal 27 xenograft tumors from 3–48 h post intravenous administration of 40 nmol 4Iphf-HN17-IR800 and HN1-IR800 peptides, and at 48 h after partial removal of the skin, which partially attenuated the fluorescence emitted from the tumors. The skinned images were obtained: left two simultaneously at 75 ms exposure time; right one: HN1-IR800 only at 300 ms exposure time. The tumor (T) and muscle (M) fluorescence images are of 2 mm slices imaged simultaneously. Other organs are in Supplementary Materials Figure S7. The bar graph shows the ratio of observed fluorescence of tumor and muscle (summed data as average with S.D.) The pairs of data marked with * are significantly (*p* < 0.05) different between HN1-IR800 and 4Iphf-HN17-IR800 2 mm slices by paired *t*-test (*n* = 4: 24, 48 h, *n* = 3: 8 h).

4Iphf-HN17-IR800 was further investigated in mice bearing Cal 27 flank xenograft tumors. Results are shown in Figure 6 with further organ images in Figure S7. We used an intravenous dose of 40 nmol per mouse (~2 μmol/kg), which appeared on a pilot study to quickly distribute throughout the tissue space. The 40 nmol dose was chosen to produce at least a 10 μM concentration when distributed into ~4 mL of extracellular space to match the 10 μM concentration used in the screening experiments. The mice were imaged at 3–48 h post administration. The IR800 dye used is known to be associated with high tissue background at earlier times [13,24,25,26], and its mass is up to 40% of the total mass of conjugates. Counter balancing this, uptake in tumors was also slow to wash out. As shown in Figure 6, tumors gradually stood out more prominently as the background fluorescent signal diminished. This was also found for UM-SCC-1 tumor mice (data not shown) and orthotopically implanted medullary thyroid tumor mice treated with 4Iphf-HN17-IR800 [27]. In contrast, tumors in mice administered 40 nmol of HN1-IR800 exhibited a similar fluorescence intensity in the tumor and in the rest of the body during the whole observation period, even at high exposure rates in the imager. Tumors with 4Iphf-HN17-IR800, but not HN1-IR800, were more conspicuous after the mouse was skinned. To better compare and semi-quantitate the fluorescence intensity, excised tumors were imaged adjacent to similarly sized masses of excised skeletal muscle. The tumor and muscle masses were then sliced into 2 mm thickness samples to minimize differential attenuation of the emitted light, as described previously [28]. Figure 6 shows that the images of tumors from 4Iphf-HN17-IR800 administered mice had greater tumor to muscle signal ratios for both whole tumor and 2 mm thickness sections at 48 h post administration, with a nearly four-fold difference between the fluorescence emission of the 2 mm thickness tumor versus muscle (*n* = 4, *p* < 0.05).

## 3. Discussion

Hong and Clayman derived HN1 from a phage display library against a single human HNSCC cell line, screening for HNSCC cell binding of the phage virus and non-binding to normal human fibroblasts. Performing cell internalization studies with HN1-FITC at 2.6 μM for 48 h, they found that: (1) HN1-FITC internalized in a time and dose dependent manner into six HNSCC cell lines but not immortalized untransformed oral epithelial cells, and not in a prostate cancer or a colon cancer cell line; (2) 200-fold excess HN1 inhibited uptake of HN1-FITC; (3) HN1-FITC stained fresh frozen unfixed human tumor tissue; (4) at a 260 nmol dose, HN1-FITC localized in vivo in mouse xenograft tumors; (5) HN1 was judged to be sequence specific based upon the negative internalization of a “jumbled” peptide, HNJ that clustered the apolar and polar amino acids [18]. Bao et al. confirmed HN1 specific uptake in further HNSCC cell lines, and found that a HN1-PKCε conjugate was internalized and blocked the activity of PKCε, inhibiting tumor growth in a xenografted mouse model [19]. Un et al. showed that HN1-anti-hRRM2, a peptide-siRNA conjugate was internalized in HNSCC and human breast carcinoma cells and suppressed expression of endogenous hRRM2 [20]. In the context of in vivo imaging agent discovery, these studies used suboptimally long incubation times (>24 h) to demonstrate reliable cell internalization of HN1. Recognizing this problem, Dudas et al. explored a wider range of conditions for HN1 binding and scrambled the peptide sequence without clustering the polar and apolar amino acids, making HNscr, which did not differ significantly in uptake from HN1. They concluded that HN1/HNscr were not very sensitive to the specific amino acid sequence, but required the long incubation periods [21].

Our attempts to reproduce the original HN1-FITC by conjugating FITC-NHS to the synthesized HN1 peptide resulted in a mixture of three peptides with FITC conjugated to either and both threonine (T) and lysine (K), as determined by MS. Details of the published syntheses of HN1-FITC and HN1-Cy5 conjugations were insufficient to conclude whether single peptides or mixtures had been used in the previously reported biological experiments. To generate discrete conjugates with the dyes conjugated to lysine, we conjugated the peptides with the FITC, Cy5-NHS, or IR800-NHS before removing the terminal Fmoc [29].

Our primary aim was to determine whether shorter incubations with HNSCC cells were enabled by increasing the lipophilicity of HN1. Fmoc addition was a convenient initial choice due to its necessity in the synthesis, and a logical one because Fmoc-peptides are known to support formation of highly hydrated scaffolds that have been used to encourage cell adhesion [30,31,32]. f-HN1 showed a greater uptake rate than HN1. The 4Iph group was chosen to add the ability to radioactively label the final molecule with radioactive iodine isotopes useful in quantitation and also in nuclear imaging. 4Iph-HN1 produced a similar effect as Fmoc seemingly proportional to its relative lipophilicity. Under the assumption that HN1 was an amphipathic CPP dependent on alternating polar and apolar amino acids, we re-sequenced HN1 to further separate the polar and apolar amino acids as in Table 1 to create HN17. f-HN17 showed faster cell uptake than f-HN1. As shown in Figure 2, combining the modifications, adding both Fmoc and 4Iph to the HN17 peptide, produced the fastest cell binding molecule, which was subsequently studied against the parent HN1 peptide as the IRDye800 conjugates.

The method development started by testing the fluorescence emission characteristics of the new molecules in different media (Figure S1). We found no obvious influence of the dye on the rate of uptake, similar to published findings on HN1-dye. The binding media contained FBS to support the viability of the cells, which led to serum protein binding (Figure 4) and some metabolism (Figure 5). If we assume that the two compounds tested, HN1-IR800 and 4Iphf-HN17-IR800, represent most of the range in the series, we can conclude that protein binding is a variable in the cellular uptake, stronger protein binding reducing cell uptake by reducing the concentration of the free peptide available to bind the cells, but that the rate determining step in the cellular uptake rate measured does not involve protein-bound peptide, since the fastest uptake rates are found for the most lipophilic and most protein bound analog, 4Iphf-HN17-IR800.

Cell uptake shown in Figure 2 correlated with lipophilicity as determined by relative HPLC Rt (Figure S6) for the lipophile enhanced peptides. Notably, the original negative control peptide analog, HNJ-IR800, was unaffected by Fmoc addition, while the Fmoc analog of the original HNSCC-specific internalizing peptide analog, f-HN1-IR800, had a significantly enhanced uptake rate compared to HN1-IR800. While 4Iph-HN1-IR800 did not reach statistical significance in uptake rate relative to HN1-IR800, 4Iph was highly effective in combination with Fmoc addition in the re-sequenced peptide, 4Iphf-HN17-IR800.

Our in vivo screening confirmed the in vitro findings on the peptides. At our 40 nmol intravenous dose used in vivo, we estimate the initial plasma concentration to be ~50 μM, and the extracellular concentration to be ≥10 μM within a few minutes of administration in mice. Hence, the peptides easily have a sufficient stability and concentration to target the tumor cells before being metabolized. It is interesting that the re-sequenced peptide, 4Iphf-HN17-IR800, had greater resistance to serum degradation than the phage-derived sequence, HN1-IR800. The latter was created in growth media containing serum specifically to ensure its ultimate stability as an in vivo delivery agent. It is possible that the stronger protein binding of 4Iphf-HN17-IR800 contributes to the serum stability by isolating a greater fraction of the HN17 peptide from serum peptidases.

The higher plasma concentration of 4Iphf-HN17-IR800 compared to HN1-IR800 is consistent with its greater serum stability and serum protein binding. The greater tumor signal and tumor to muscle ratio of 4Iphf-HN17-IR800 is probably a result of multiple factors: (1) Cal 27 cell uptake of 4Iphf-HN1-IR800 is much faster and achieves a greater peak concentration; (2) blood clearance is slower and serum stability greater for 4Iphf-HN17-IR800, exposing the tumor to the intact agent for a longer time; (3) molar fluorescence emission of 4Iphf-HN17-IR800 is approximately 1.5-fold greater than HN1-IR800 when serum proteins are present. These factors outweighed the stronger serum protein binding of 4Iphf-HN17-IR800 that would probably reduce the bioavailability of the unbound peptide for tumor uptake in vivo.

The present work had the purpose of creating a useful HNSCC agent for clinical Optical Surgical Navigation (OSN). We used the NIRF dicyanine, IRDye800 (IR800), which emits in the optimal ≥800 nm range detectable with FDA approved optical imagers [33]. This dye also has a published positive toxicologal profile [34,35]. Uptake, as we use the term, probably includes a rapid adherence to the cell membranes followed by a rate determining internalization step, but the repeated PBS washing probably did not remove all of the membrane bound molecules, so the signal detected in the uptake rate studies is the sum of the fluorescent signal from the internalized and residual membrane bound molecules.

By operational definition, the HN1 peptides have been classified as hydrophobic Cell Penetrating Peptides (CPP), the hydrophobic examples being the rarest and least understood among the approximately 1800 CPP [22,23]. The CPP functionality is typically at micromolar concentration, without classical receptor specificity, and they have the ability to internalize in cells, usually into endosomes, and carry cargo, usually therapeutic drugs covalently conjugated. CPP have been divided into three classes. One class is derived from protein transduction domains (e.g., TAT) or analogs (poly-Arginine’s) that are always cationic, and internalize through various types of pinocytosis. A second class known as amphipathic CPP form helical wheels and are able to penetrate through cell membranes directly [36,37]. A third class are hydrophobic and tend to lack cationic charges.

The new peptide compounds represented by 4Iphf-HN17-IR800, with additional non-peptidic hydrophobic elements and overall anionic charges, are an unusual molecular type of CPP lacking a strong dependence on overall charge. CPP also do not typically show such strong cancer cell specificity as HN1 did, although we have not verified that this feature of HN1 is retained in the HN17 peptide analogs. CPP have been studied for over 25 years, with very little practical success at therapeutic drug delivery due to poor ability to escape from endosomes after internalization [38,39], but for imaging applications, endosomal escape is probably unnecessary.

### Limitations of the Study

Caveats of our study include that we have not demonstrated that HN1’s cancer specificity is maintained in the new peptides, if it indeed exists. Additionally, the internalization mechanism(s) of uptake of the new molecules remains to be explored. Further work beginning with these two issues, and extending to toxicity studies, will be required to refine our understanding of these new molecules and the scope of their usefulness.

## 4. Materials and Methods

Resins, reagents, and all amino acids were purchased either from AAPPTEC Inc (Louisvelle, KY, USA) or CHEMIMPEX International Inc. Wood Dale, IL, USA)). The solvents for the syntheses and purifications were procured from Greenfield Global Inc. (Brookfield, CT, USA) at reagent grade. The peptides were assembled using an Endeavor 90 Solid Phase Peptide Synthesizer manufactured by AAPPTEC Inc.

Peptide conjugate molecules were synthesized in two stages. The starting peptide molecules were procured using solid phase peptide synthesis with standard Fmoc protection strategy. Thus, for each mmol of the amine on the resin, 4.0 mmol of protected amino acid was activated for 5 min with 4.0 mmol of the appropriate coupling agent HATU (Hexafluorophosphate Azabenzotriazole Tetramethyl Uronium) or HBTU (Hexafluorophosphate Benzotriazole Tetramethyl Uronium) and 8.0 mmol of *N*,*N*-Diisopropylethylamine (DIEA). Then the activated acid was transferred to the amine on the solid phase and the reaction vessel was shaken for 1 h. The final products and the protection groups were released from the resin using a 10 mL solution of trifluoroacetic acid (TFA), phenol, tris-isopropylsilane (TIPS), and water in a ratio of 95:2:2:1 (process repeated twice). Then the mixture was precipitated into methyl-*tert*-butyl ether. The precipitate was filtered and the crude solid was purified on preparative HPLC (Shimadzu preparatory purification unit (LC8A)) using a C18 column (10 μm, 50 × 250 mm, 60 min runtime at 100 mL/min) with a gradient of water (0.1% TFA): acetonitrile (MeCN) (0.1% TFA) between 10% and 100%. Fractions with >90% purity were pooled and checked for the product by MS (mass spectral analysis). The fractions with the required mass and purity >90% were pooled and freeze dried (AAPTEC Sharp Freeze-110) to yield the product as a colorless fluffy solid.

The preparation of HN17 required that the final coupling be performed by replacing amino acid N-Fmoc-O-*tert*-butyl-L-threonine by Fmoc-4-Iodo-L-Phenylalanine. The removal of Fmoc protection in the dye conjugates f-HN-1-IR800 and f-HNJ-IR800 to afford HN1-IR800 and HNJ-IR800 was accomplished by incubating the molecules with 20% diethyl amine in MeCN at ambient temperature. The reaction was monitored by HPLC and was terminated by evaporation under vacuum that was followed by HPLC purification to afford the desired product. Some loss of final product due to decomposition occurs during removal of the Fmoc, but the decomposition product has a Rt (HPLC retention time) several minutes different than the final product, and so was simple to eliminate via preparative HPLC.

Fluorescent labeling. To an equimolar amount (0.65 μmol) of the purified peptide and IR800-NHS ester in DMSO (dry, 250 μL) 4-methyl morpholine (5 μL) was added. The resultant mixture was incubated at 40 °C for 1 h. After the completion of the reaction (ascertained by LC/MS, MALDI) the product was isolated by preparative HPLC on a Sunfire (Waters) C18 (30 × 250 mm, 5 μm) column with 30 mL/min flow rate. The solvent system consisted of the water (0.1% TFA) and MeCN (acetonitrile) (0.1% TFA) with MeCN ascending from 5% to 70% over 60 min. After the analysis, the final compound was collected and lyophilized to afford a greenish blue product in approximately 90% purity.

High resolution MS scans were used to confirm the product identity. Table S1 contains the analytical data on final molecules. For each synthesized dye conjugate peptide molecule, we used matrix assisted laser desorption/ionization time-of-flight (MALDI-TOF) performed on a Bruker Daltonics UltrafleXtreme™ (Bruker Daltonics, Breman, Germany) mass spectrometer operated in reflection, positive ion mode with a N_2_ smartbeam II™ laser (337 nm). Laser power was used at the threshold level required to generate signal and acquired at 1000 Hz until suitable data were obtained. The instrument was calibrated with the Peptide Calibration Standard II purchased from Bruker Daltonics which contains Angiotensin II, Angiotensin I, Substance P, Bombesin, ACTH clip 1-17, ACTH clip 18-39, Somatostatin 28, Bradykinin Fragment 1-7, Renin Substrate Tetradecapeptide porcine with a covered mass range: ~700–3200 Da.

Analysis of the purity (>90%) of the synthesized products and starting peptides was performed using a Shimadzu LC-10ATvp model HPLC, and a Waters C18-RP analytical column (XBridge cartridge, 150 × 4.6 mm, 3.5 μm; flow rate = 1 mL/min) starting at 80:20 water (0.1% TFA): MeCN (0.1% TFA) for the first 10 min and then a linear gradient over 20 min up to 70% MeCN. The HPLC peaks for dye-conjugated products were visualized with a fluorescence detector (RF-10AXL, Shimadzu) detecting NIRF emission and the purity was in each case >90% by relative HPLC peak area at 750–820 nm. The starting peptide peaks were visualized with a UV-Vis detector (254 nm) and the purity was >90% by relative HPLC peak area. Table S1 contains the retention time data, which was also used as a measure of lipophilicity.

Human oral squamous carcinoma cell line, Cal 27, was purchased from American Type Culture Collection (ATCC, CAT # CRL-2095) and maintained at 37 °C with 5% CO_2_ in DMEM supplemented with 10% fetal bovine serum (FBS) and 100 U penicillin/streptomycin. Cells were passaged twice per week.

Cell uptake. Cal 27 cells were seeded at 7000–12,000/well in a 96-well plate in triplicate for each reaction. To determine proper incubation duration, the medium was replaced after 24–48 h with 150 μL of medium per well containing 0–50 μM HN peptides and incubated for 2–48 h. For comparative uptake rate experiments, the medium was replaced after 24 h with 150 μL of medium per well containing 0–10 μM HN peptides and incubated for 1 or for 2 h. Cells were then washed five times with 150 μL of PBS. PBS was removed completely after the last wash and cells were lysed in 60 μL lysis buffer (62.5 mM Tris-HCl at pH = 6.8, 2% SDS, and 10% glycerol). Fluorescence intensity was measured using a BioTek Synergy H4 plate reader with ex/em at 764/809 nm for IR800-conjugates, 677/735 nm for Cy5-conjugates and 485/528 nm for FITC-conjugates. Measurements were made in the presence of BSA on the lysed preparations (Figure S1). To normalize data, a single peptide, f-HN1-IR800, was included in all of the Cal 27 cell screening experiments for newly developed agents and readings of f-HN1-IR800 at 10 μM were set as 100%. Cell numbers were controlled by a duplicate plate with the same treatment. Duplicate independent experiments were performed and the cells were counted on the duplicates. A *t*-test was used to analyze the 10 μM, 2 h data.

Fluorescence microscopy assays. Cal 27 cells were seeded at 70,000/well in duplicate on eight-well chamber slides and allowed to attach overnight. Cell culture medium was replaced with 200 μL medium containing 10 μM peptides. Cells were incubated at 37 °C for 1–24 h followed by washing four times with 300 μL of HEPES buffer (25 mM HEPES 150 mM NaCl at pH 7.4) and once with the buffer containing 1 μg/mL DAPI. The chamber slide scaffold was then removed. Each chamber was then covered by a drop of aqua-poly mount and a coverslip, and sealed with clear nail polish. Cells were imaged with an Olympus IX81 microscope using an 800 nm emission filter set for IRDye800 conjugates and 461 nm for DAPI. Images were captured at 800 nm (red) for 5 s and at 461 nm (blue) with the automatic settings, at 200×.

To visualize HNSCC cell internalization of the peptides, we used the 4Iphf-HN17-Cy5 conjugate to remain within the upper emission range of the Olympus Fluoview FV10iCal 27 confocal laser scanning microscope (Ex. 647 nm/Em. 665 nm) with a 60× objective. Cal 27 cells were seeded at a density of 1 × 10^6^ cells and incubated in medium for 16 h. The cells were then incubated with 5 µM 4Iphf-HN17-Cy5 for 1.5 h, then washed with DPBS (Dulbecco’s phosphate-buffered saline), Hoechst 33,342 was added (10 min) to stain nuclei, and then growth medium was added for confocal imaging. 4Iphf-HN17-FITC was shown to be >90% cell associated by FACS (BD FacsCanto II). In the blocking experiment, the Cal 27 cells were first incubated with 100 µM 4Iphf-HN17 for 1.5 h, washed with DPBS, then treated as above.

FBS protein binding assay. 4Iphf-HN17-IR800 and HN1-IR800 (final concentration 25 μM) were incubated in 400 μL of FBS at room temperature for 30 s. The concentration was chosen as the maximum expected in blood from an intravenous imaging dose of 40 nmol in a 20 g mouse, with a blood factor of 0.078 [40,41]. Solutions (300 μL) were then loaded into an Amicon unit (0.5 mL, 10 K cutoff) and centrifuged at 12,000× *g* for 15 min [28]. Samples from the filtrate (50 μL), residual (5 μL), and original solution (5 μL) were loaded in duplicate into black wall 96-well plates containing 50 (for filtrate) or 95 (for residual and original) μL of PBS with 0.2% BSA. For an additional wash, 300 μL of PBS was then added into the Amicon unit. The unit was centrifuged as above. The same amounts of each of the parts were loaded into wells. Fluorescence intensity for each fraction was calculated by fraction volume × fluorescent unit/μL. Single factor ANOVA was used to analyze the data with *p* < 0.05 considered significant.

In vitro serum stability. Agents 4Iphf-HN17-IR800 and HN1-IR800 (final concentration 6.4 μM) were incubated in 200 μL of fresh mouse serum (from nu/nu mice) at 37 °C for 0, 0.5, 1.5, 3, and 6 h. Agents were separated from serum proteins by addition of 2% of SDS as described previously [28], followed by mixing with 100 μL ice-cold ETOH and 300 μL of ACN, and centrifuged at 12,000× *g* for 20 min at 4 °C. The liquid phase (50 μL) was analyzed by HPLC with a C18 reversed phase column detected via 800 nm emission fluorescence using a Shimadzu RF-10AXL fluorescence HPLC detector. Separate control samples values included buffer only, 4Iphf-HN17-IR800 and HN1-IR800, and IRdye800-CW. Relative quantities of agents over time were determined based on the peak areas in the chromatograms. A degradation curve was fit, and a half-life calculated using MS Excel.

Blood clearance. Normal female Balb/c mice, 6- to 8-week old, were used. Blood samples (5 μL) were collected from the saphenous vein at 2 min, 0.5, 1, 3, 6, and 24 h post-injection (p.i.), and loaded into a 96-well plate with each well containing 95 μL of PBS with 0.15% EDTA (pH 8.5) and 0.2% BSA. Mice urine was collected until 3 h post injection without hydrating the mice. For urine accumulation analysis, 1 μL of urine was loaded into the 96-well plate in triplicate and diluted with 99 μL of PBS. Blood and urine samples from an uninjected mouse were used as negative controls. Fluorescence intensity was measured using the BioTek Synergy H4 plate reader. The total blood fluorescence (% ID/blood) (ID is injected dose) for each mouse was calculated as (blood volume × fluorescent unit per μL blood)/(fluorescent unit per μL ID × 100), and 3 h urine excretion for each mouse was calculated as (% ID/urine) equals (urine volume × fluorescent unit/μL urine)/(fluorescent unit per μL ID × 100). Blood volume was calculated based upon mouse weight [40]. Fluorescence change in blood was plotted versus time. All data are presented as mean (SD). A Student’s *t*-test was employed to analyze the difference between two points. A *p* value of 0.05 was considered to be statistically significant.

In vivo imaging. All experiments using live animals were conducted in accordance with protocols approved by The Ohio State University Institutional Animal Care and Use Committee (protocol 2010A0056). Female nude mice (nu/nu), 5–7 week old, were purchased from Charles River. Cal 27 cells (1.5 × 10^7^) in 100 µL PBS were inoculated subcutaneously in the left flank. Tumor size was measured twice a week and the volume was calculated using a formula: length × width × width/2. The diet for mice was shifted from regular to fluorescence reduced (CAT# TD.97184, Harlan, WI) chow 1 week before imaging.

When the tumors grew approximately to 150 mm^3^, the mice were injected via tail vein with 40 nmol of 4Iphf-HN17-IR800 or HN1-IR800 in 100 µL of PBS with 10% DMSO. The animals were imaged using a laser excitation Fluobeam^TM^ 800 NIR imaging system (Fluoptics, Grenoble, France) as published previously [28]. Imaging was performed on whole and euthanized, skinned mice, whole excised tumors alongside similar sized muscle chunks, and on 2 mm tumor and muscle slices. The fluorescence intensities of the tumors were compared quantitatively to their muscle controls (all as 2 mm slices) using Image J software to calculate relative ratios from the image intensities.

## 5. Conclusions

Starting with a FITC-labeled, phage-derived peptide, HN1-FITC, resequencing it, substituting N-terminal lipophiles, and labeling with a practical NIRF dye produced 4Iphf-HN17-IR800. This new optical agent has much more rapid tumor cell uptake, greater and more useful fluorescence emission, and improved in vivo tumor imaging characteristics. These features qualify it for further investigation as a practical imaging tool for OSN in HNSCC. The new compounds behave like amphipathic CPP, rapidly internalizing to the cytosol in HNSCC cells. Given the positive tumor targeting characteristics of 4Iphf-HN17-IR800, there is reason to expect improved effectiveness on substituting 4Iphf-HN17 for HN1 in targeted imaging and therapeutic drugs.

## Figures and Tables

**Table 1:** Compound names and peptide sequences as single letter codes. Polar amino acids are in bold. f = Fmoc; 4Iph = 4-para-iodo-benzyl for HN17 series, and 4-para-iodo-benzoyl for HN1 series. Dyes are conjugated to lysine (K).

Name	Sequence
HN1	**TS** *PL* **N** *I* **HN** *G* **QK*L***
f-HN1	f**-TS***PL***N***I***HN***G***QK***L*
4Iph-HN1	4Iph**-TS***PL***N***I***HN***G***QK***L*
HNJ	**NQHSKNT** *LLIGP*
f-HNJ	f**-NQHSKNT***LLIGP*
HNscr	*L* **NKQTH** *GLIP* **NS**
f-HN17	f-**T**LP**NSNH***I***KQ**G**L**
4Iphf-HN17	(4Iph)-f-*LP***NSNH***I***KQ***G****L***

## Supplementary Materials

The following are available online at https://www.mdpi.com/1420-3049/24/17/3070/s1, Figure S1: NIRF signal vs. concentration and FBS for peptides. Figure S2: Cal 27 uptake of peptide in lysed vs. unlysed cells. Figure S3: Cell uptake of peptides at 1 and 2 h incubations. Figure S4: Fluorescent microscopy showing uptake of 4Iphf-HN17-IR800 in Cal 27 cells. Figure S5: Blood clearance and urine accumulation of peptides in mice. Table S1: HPLC Purity, mass spectral identity, HPLC lipophilicity, and cell uptake of peptides. Figure S6: Plot of Cal 27 cell uptake vs. lipophilicity of peptides. Figure S7: Fluobeam optical images of normal organs after peptide administration. Table S2: Data set used to generate fluorescent intensities in uptake experiments. Table S3: Numerical data plotted in Figure 2 with uncertainty and *p* values.

## References

[1] https://www.cancer.gov/research/progress/snapshots/head-and-neck.

[2] Arbes S.J., Olshan A.F., Caplan D.J., Schoenbach V.J., Slade G.D., Symons M.J. (1999). Factors contributing to the poorer survival of black Americans diagnosed with oral cancer (United States). Cancer Causes Control.

[3] Vokes E.E., Weichselbaum R.R., Lippman S.M., Hong W.K. (1993). Head and neck cancer. N. Engl. J. Med..

[4] Eldeeb H., Macmillan C., Elwell C., Hammod A. (2012). The effect of the surgical margins on the outcome of patients with head and neck squamous cell carcinoma: Single institution experience. Cancer Biol. Med..

[5] Woolgar J.A., Triantafyllou A. (2005). A histopathological appraisal of surgical margins in oral and oropharyngeal cancer resection specimens. Oral Oncol..

[6] Rosenthal E.L., Kulbersh B.D., Duncan R.D., Zhang W., Magnuson J.S., Carroll W.R., Zinn K. (2006). In vivo detection of head and neck cancer orthotopic xenografts by immunofluorescence. Laryngoscope.

[7] Potala S., Verma R.S. (2011). Targeting head and neck squamous cell carcinoma using a novel fusion toxin-diphtheria toxin/HN-1. Mol. Biol. Rep..

[8] Rosenthal E.L., Warram J.M., de Boer E., Chung T.K., Korb M.L., Brandwein-Gensler M., Strong T.V., Schmalbach C.E., Morlandt A.B., Agarwal G. (2015). Safety and Tumor Specificity of Cetuximab-IRDye800 for Surgical Navigation in Head and Neck Cancer. Clin. Cancer Res..

[9] Newman J.R., Gleysteen J.P., Baranano C.F., Bremser J.R., Zhang W., Zinn K.R., Rosenthal E.L. (2008). Stereomicroscopic fluorescence imaging of head and neck cancer xenografts targeting CD147. Cancer Biol. Ther..

[10] Day K.E., Sweeny L., Kulbersh B., Zinn K.R., Rosenthal E.L. (2013). Preclinical comparison of near-infrared-labeled cetuximab and panitumumab for optical imaging of head and neck squamous cell carcinoma. Mol. Imaging Biol..

[11] James M.L., Gambhir S.S. (2012). A molecular imaging primer: Modalities, imaging agents, and applications. Physiol. Rev..

[12] Harlaar N.J., Kelder W., Sarantopoulos A., Bart J., Themelis G., van Dam G.M., Ntziachristos V. (2013). Real-time near infrared fluorescence (NIRF) intra-operative imaging in ovarian cancer using an alpha(v)beta(3)-integrin targeted agent. Gynecol. Oncol..

[13] Shrivastava A., Ding H.M., Kothandaraman S., Wang S.H., Gong L., Williams M., Milum K., Zhang S., Tweedle M.F. (2014). A High-Affinity Near-Infrared Fluorescent Probe to Target Bombesin Receptors. Mol. Imaging Biol..

[14] Wright C.L., Pan Q., Knopp M.V., Tweedle M.F. (2016). Advancing theranostics with tumor-targeting peptides for precision otolaryngology. World J. Otorhinolaryngol. Head Neck Surg..

[15] Hsiao J.R., Chang Y., Chen Y.L., Hsieh S.H., Hsu K.F., Wang C.F., Tsai S.T., Jin Y.T. (2010). Cyclic alpha v beta 6-targeting peptide selected from biopanning with clinical potential for head and neck squamous cell carcinoma. Head Neck-J. Sci. Spec..

[16] Nothelfer E.M., Zitzmann-Kolbe S., Garcia-Boy R., Kramer S., Herold-Mende C., Altmann A., Eisenhut M., Mier W., Haberkorn U. (2009). Identification and characterization of a peptide with affinity to head and neck cancer. J. Nucl. Med. Off. Publ. Soc. Nucl. Med..

[17] Atallah I., Milet C., Henry M., Josserand V., Reyt E., Coll J.L., Hurbin A., Righini C.A. (2016). Near-infrared fluorescence imaging-guided surgery improves recurrence-free survival rate in novel orthotopic animal model of head and neck squamous cell carcinoma. Head Neck.

[18] Hong F.D., Clayman G.L. (2000). Isolation of a peptide for targeted drug delivery into human head and neck solid tumors. Cancer Res..

[19] Bao L., Gorin M.A., Zhang M., Ventura A.C., Pomerantz W.C., Merajver S.D., Teknos T.N., Mapp A.K., Pan Q. (2009). Preclinical development of a bifunctional cancer cell homing, PKCepsilon inhibitory peptide for the treatment of head and neck cancer. Cancer Res..

[20] Un F., Zhou B., Yen Y. (2012). The utility of tumor-specifically internalizing peptides for targeted siRNA delivery into human solid tumors. Anticancer Res..

[21] Dudas J., Idler C., Sprinzl G., Bernkop-Schnuerch A., Riechelmann H. (2011). Identification of HN-1-Peptide Target in Head and Neck Squamous Cell Carcinoma Cells. ISRN Oncol..

[22] Milletti F. (2012). Cell-penetrating peptides: Classes, origin, and current landscape. Drug Discov. Today.

[23] Agrawal P., Bhalla S., Usmani S.S., Singh S., Chaudhary K., Raghava G.P.S., Gautam A. (2016). CPPsite 2.0: A repository of experimentally validated cell-penetrating peptides. Nucleic Acids Res..

[24] Choi H.S., Gibbs S.L., Lee J.H., Kim S.H., Ashitate Y., Liu F.B., Hyun H., Park G., Xie Y., Bae S. (2013). Targeted zwitterionic near-infrared fluorophores for improved optical imaging. Nat. Biotechnol..

[25] Tweedle M.F., Ding H.M., Drost W.T., Dowell J., Spain J., Joseph M., Elshafae S.M., Menendez M.I., Gong L., Kothandaraman S. (2018). Development of an orthotopic canine prostate cancer model expressing human GRPr. Prostate.

[26] Lantry L.E., Cappelletti E., Maddalena M.E., Fox J.S., Feng W., Chen J., Thomas R., Eaton S.M., Bogdan N.J., Arunachalam T. (2006). 177Lu-AMBA: Synthesis and characterization of a selective 177Lu-labeled GRP-R agonist for systemic radiotherapy of prostate cancer. J. Nucl. Med..

[27] Rossfeld K.K., Justiniano S.E., Ding H.M., Gong L., Kothandaraman S., Sawant D., Saji M., Wright C.L., Kirschner L.S., Ringel M.D. (2017). Biological Evaluation of a Fluorescent-Imaging Agent for Medullary Thyroid Cancer in an Orthotopic Model. J. Clin. Endocrinol. Metab..

[28] Ding H., Kothandaraman S., Gong L., Williams M.M., Dirksen W.P., Rosol T.J., Tweedle M.F. (2016). A human GRPr-transfected Ace-1 canine prostate cancer model in mice. Prostate.

[29] Carpino L.A., Han G.Y. (1972). 9-Fluorenylmethoxycarbonyl amino-protecting group. J. Org. Chem..

[30] Castelletto V., Moulton C.M., Cheng G., Hamley I.W., Hicks M.R., Rodger A., López-Pérez D.E., Revilla-López G., Alemán C. (2011). Self-assembly of Fmoc-tetrapeptides based on the RGDS cell adhesion motif. Soft Matter.

[31] Gong X., Branford-White C., Tao L., Li S., Quan J., Nie H., Zhu L. (2016). Preparation and characterization of a novel sodium alginate incorporated self-assembled Fmoc-FF composite hydrogel. Mater. Sci. Eng. C Mater. Biol. Appl..

[32] Jayawarna V., Richardson S.M., Hirst A.R., Hodson N.W., Saiani A., Gough J.E., Ulijn R.V. (2009). Introducing chemical functionality in Fmoc-peptide gels for cell culture. Acta Biomater..

[33] Rosenthal E.L., Warram J.M., de Boer E., Basilion J.P., Biel M.A., Bogyo M., Bouvet M., Brigman B.E., Colson Y.L., DeMeester S.R. (2016). Successful Translation of Fluorescence Navigation During Oncologic Surgery: A Consensus Report. J. Nucl. Med..

[34] Marshall M.V., Draney D., Sevick-Muraca E.M., Olive D.M. (2010). Single-dose intravenous toxicity study of IRDye 800CW in Sprague-Dawley rats. Mol. Imaging Biol..

[35] Zinn K.R., Korb M., Samuel S., Warram J.M., Dion D., Killingsworth C., Fan J., Schoeb T., Strong T.V., Rosenthal E.L. (2015). IND-directed safety and biodistribution study of intravenously injected cetuximab-IRDye800 in cynomolgus macaques. Mol. Imaging Biol..

[36] Heitz F., Morris M.C., Divita G. (2009). Twenty years of cell-penetrating peptides: From molecular mechanisms to therapeutics. Br. J. Pharm..

[37] Guidotti G., Brambilla L., Rossi D. (2017). Cell-Penetrating Peptides: From Basic Research to Clinics. Trends Pharm. Sci..

[38] Qian Z., Martyna A., Hard R.L., Wang J., Appiah-Kubi G., Coss C., Phelps M.A., Rossman J.S., Pei D. (2016). Discovery and Mechanism of Highly Efficient Cyclic Cell-Penetrating Peptides. Biochemistry.

[39] Pei D., Buyanova M. (2018). Overcoming Endosomal Entrapment in Drug Delivery. Bioconjug. Chem..

[40] Altman P.L. (1954). Blood and Other Body Fluids.

[41] Durbin P.W., Jeung N., Kullgren B., Clemons G.K. (1992). Gross Composition and Plasma and Extracellular Water Volumes of Tissues of a Reference Mouse. Health Phys..

