# Supplementary material for: Novel Peptide NIRF Optical Surgical Navigation Agents for HNSCC
Source: Molecules. 2019 Aug 23;24(17):3070. doi: 10.3390/molecules24173070 (PMC6749330; doi:10.3390/molecules24173070)
Supplement: Supplementary file 1 [file molecules-24-03070-s001.pdf]

# Supplementary Material

Novel ~~hybrid p~~Peptide NIRF Optical Surgical Navigation Agents for HNSCC


Haiming Ding[1]*, Shankaran Kothandaraman[1]*, Li Gong[1]*, Chadwick L. Wright[1]*, Quintin Pan[2], Theodore Teknos[2], Michael F. Tweedle[1]

*contributed equally to this work
[1] The Ohio State University, Department of Radiology, The Wright Center of Innovation in Biomedical Imaging, Columbus, Ohio
[2] Current address: Seidman Cancer Center, University Hospitals, Cleveland, Ohio

Corresponding Author: Michael F. Tweedle, PhD
michael.tweedle@osumc.edu
telephone: 614-247-4427


## Materials and Methods.

In development of the methods for measuring cell uptake by the ~~hybrid~~ peptides, we were aware of the difficulties inherent in using fluorescence emission intensity (NIRF) to make quantitative comparisons. This is in part due to changes in the chemical form of the molecules containing the dye while they are accumulated at the cell membrane or interior of cells, whether protein bound or free, or chemically associated in solution. We saw up to 46 % range of the NIRF measured among the IR800-conjugated derivatives in PBS with serum albumin added, and up to ~30% % increase in NIRF in the presence of FBS or BSA (Figure S1(a)(b).

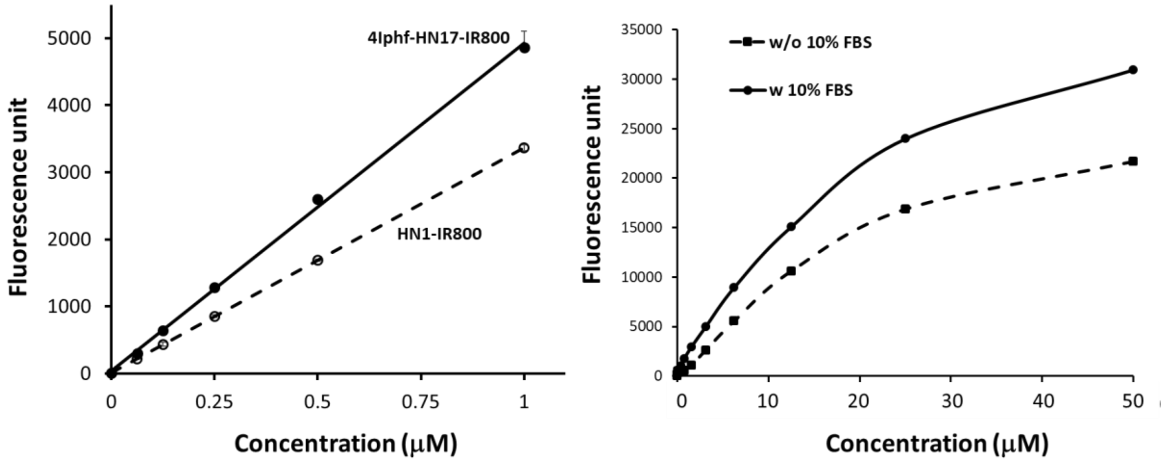


**Figure S1 Left**: linear regression fit of the NIRF signal measured on the Synergy 4 plate reader as described in the Materials and Methods section. Data for 4Iphf-HN17-IR800 (solid line) and HN1-IR800 (broken line) were measured in 0.2% bovine serum albumin (BSA) in PBS solutions from concentrations

of 0.0625 – 1 μM. The fluorescence emission of 4Iphf-HN17-IR800 was 1.46 times greater than HN1-IR800, as measured by the relative slopes. **Right:** In PBS significant loss of the fluorescence emission was observed on conjugating IR800-CW to the ~~hybrid~~ peptides (up to 47 % for 4Iphf-Hn17-IR800). Any source of albumin raised the molar NIRF intensity of all agents, even unconjugated dyes. The data in the graph demonstrate the increase in NIRF emission of adding 10% FBS to 4IpfHN17-IR800. Hence, all comparative plate reader measurements were made in the presence of equivalent amounts of albumin (BSA).

The differences observed were far lower than the differences measured among the ~~hybrid~~ peptides in the cell binding experiments shown in Figure 2. We chose to make all NIRF measurements in the presence of albumin and to lyse cells before quantitative measurements of the NIRF. Lysing raised the NIRF signal relative to the corresponding unlysed measurement, but the order of the ~~hybrid~~ peptides rate of uptake in Cal 27 cells was not affected by lysing. (Figure S2).

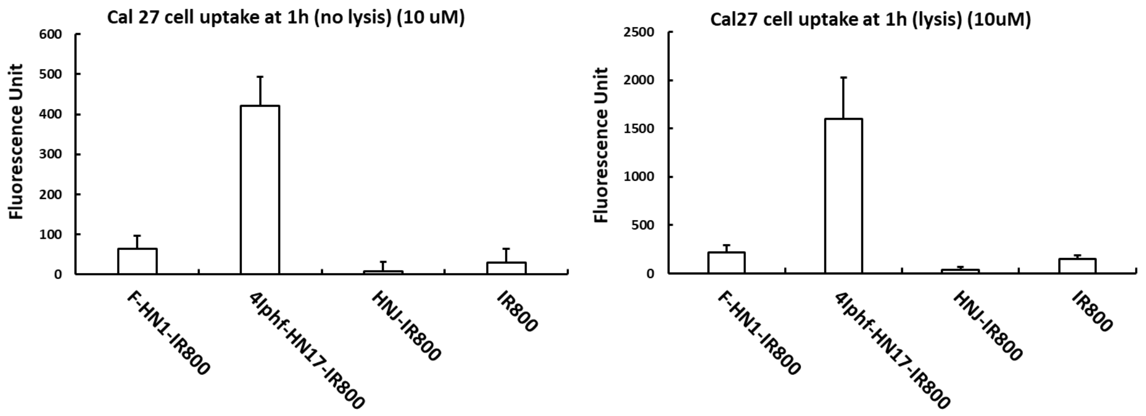


**Figure S2.** Cal-27 cell uptake retained after five PBS washes, comparing lysed and unlysed cells. Lysing cells prior to measurement on fluorescence following incubation with the ~~hybrid~~ peptides did not affect

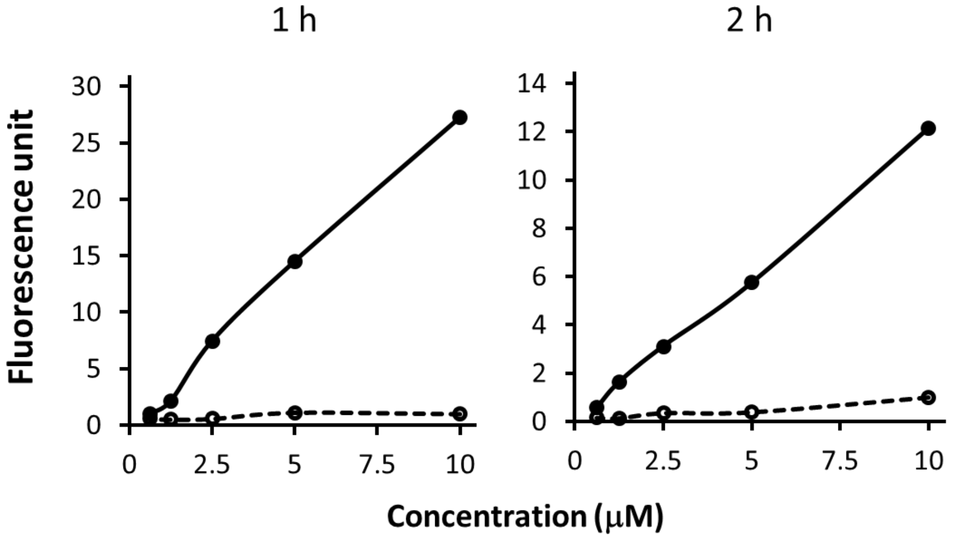

the measured fluorescence.

**Figure S3.** Comparison of the uptake into Cal-27 cells at 1 and 2 h of HN1-IR800 and 4Iphf-HN17-IR800, studied as in Figure 2, with the data normalized to the HN1-IR800. The 1 h data shows the highest ratio of 4IphfHN17-IR800 over HN1-IR800, a ratio of 27 : 1. The ratio of uptake at 2 h drops to about 13. Note: Figure 2 data are normalized to fHN-1-IR800 that was run adjacent to each of the ~~hybrid~~-peptides. f-HN1-IR800 has roughly three times the rate of uptake of HN1-IR800, which accounts for the difference in the vertical scale magnitude of 4IphfHN17-IR800 between Figure 2 at ~4.5 and Figure S3 at ~13.

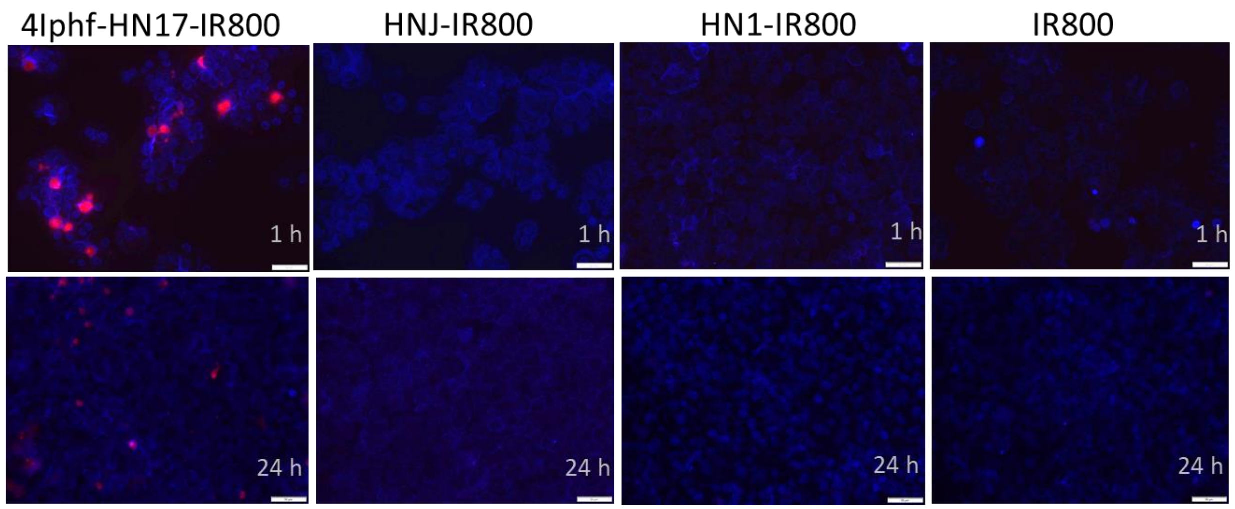


**Figure S4**.Fluorescence microscopy of 1 h and 24 h incubations of 10 µM IR800-labeled ~~hybrid~~-peptides in Cal 27 cells. 4Iphf-HN17-IR800 labels the cells strongly at 1 h, and less strongly at 24 h. HN1-IR800, HNJ-IR800, and IR800 are not observably taken up.

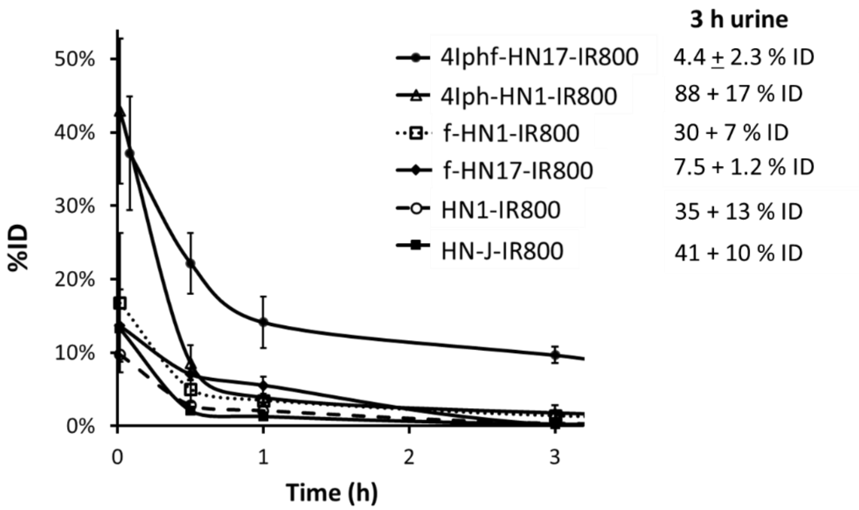


**Figure S5**. Blood clearance of ~~hybrid~~-peptide molecules from normal mice as described in Materials and Methods. Urine accumulations paralleled the blood clearance, with the 4Iph substitution producing

somewhat longer blood clearance and lower 3 h urine accumulations. Mice were not hydrated during the 3 h interval, which probably decreased blood clearance and urinary excretion over what would be measured in well hydrated mice.

**Table S1**. Analytical data for identity of the compounds, HPLC Rt values on C-18 columns run as described in materials and methods to establish purity and relative lipophilicity, run under identical conditions on the same instrument and column. Purity was assessed by HPLC single peaks to be > 90 % by peak area of the NIRF at the Rt in the table. Rt values reflect relative lipophilicity. The overall correlation coefficient was only 0.68 for Rt vs uptake

| ~~Hybrid~~ Peptide | HPLC Rt, min. * | Cal-27 uptake @2h, 10μM** | formula | MW (Calc'd) | MW (found)*** |
|---|---|---|---|---|---|
| 4Iphf-HN17-IR800 | 15.5 | 4.5 | $C_{123}H_{161}IN_{21}O_{32}S_4$ | 2698.95 | 2699.15 |
| f-HN17-IR800 | 13.8 | 1.7 | $C_{118}H_{160}N_{21}O_{33}S_4$ | 2527.04 | 2527.22 |
| f-HN1-IR800 | 13.6 | 1.0 | $C_{118}H_{160}N_{21}O_{32}S_4$ | 2527.04 | 2527.06 |
| 4Iph-HN1-IR800 | 12.4 | 0.56 | $C_{110}H_{153}IN_{21}O_{32}S_4$ | 2534.89 | 2534.98 |
| f-HNJ-IR800 | 12.7 | 0.51 | $C_{118}H_{160}N_{21}O_{32}S_4$ | 2527.04 | 2527.21 |
| HN1-IR800 | 7.31 | 0.37 | $C_{103}H_{150}N_{21}O_{31}S_4$ | 2304.97 | 2304.83 |
| HNJ-IR800 | 11.0 | 0.32 | $C_{103}H_{150}N_{21}O_{31}S_4$ | 2304.97 | 2305.05 |

* Solvent: start 20 to 50%ACN up to 10 min., 50 to 70% from 10 to 30 min. ** the highest concentration point in Figure 2. *** By MALDA-TOF mass spectral analysis as reported in Materials and Methods.

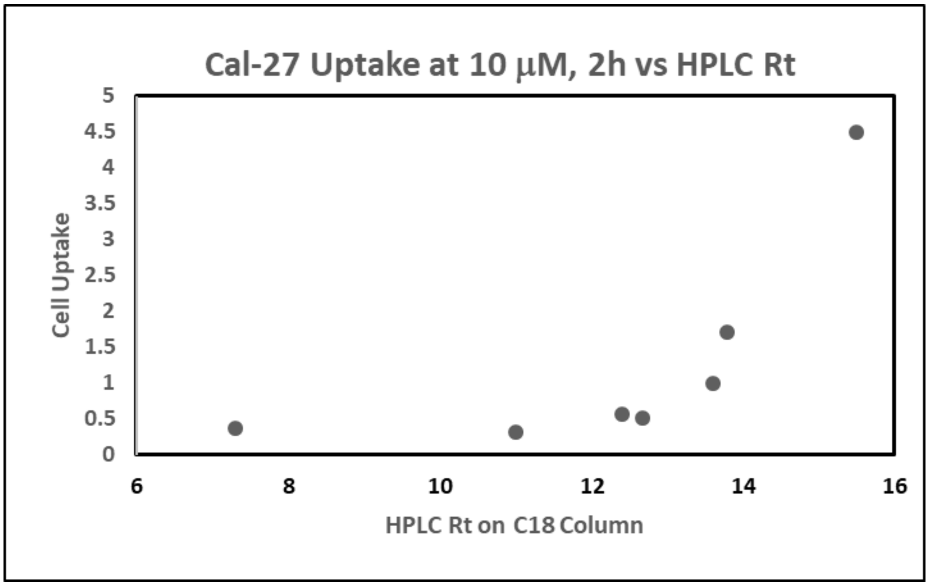


**Figure S6**. Plot of the shaded data in Table S2: HPLC Rt vs cell uptake. The data suggest some effectiveness of the lipophilic elements added to the N-terminus of the peptides that differed significantly in uptake from HN1-IR800 (the top three in uptake).

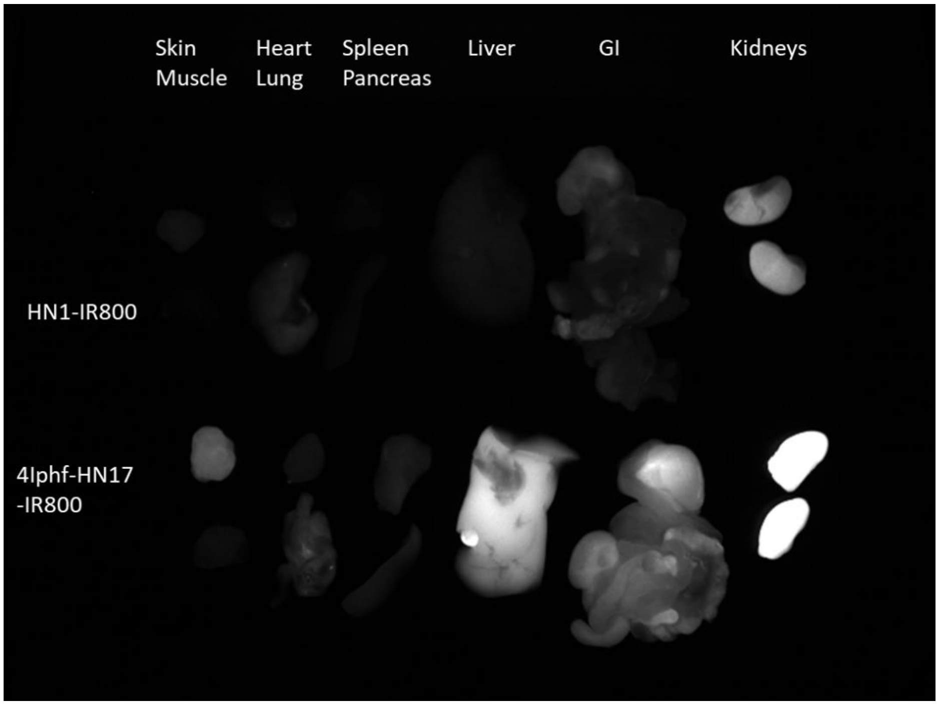


**Figure S7.** Fluobeam images of mouse organs 4 h post 40 nmol dose in mice. 4Iphf-HN17-IR800 is retained longer in excretory organs and skin. Images are typical of other mice euthanized at 24 h. The metabolic half life in mouse serum was 5.29 h (Figure 5). Hence the 800 nm emission imaged at 24 h was probably a metabolite containing the IRDye800 fragment.

**Table S2.** A data set used to generate the 10 uM Fluorescence Intensity point for f-HN17-IR800 in Figure 2 using the protocol in Materials and Methods. Each datum in the first three columns is the fluorescence intensity measured on the Syngery IV plate reader for a single well of cells. f-HN1-IR800 was run simultaneously as a control with this, and each of the other analogs reported in Figure 2 to obtain a ratio of the Analog / f-HN1-IR800. In this example that ratio was 1.45. A second independent, identical experiment gave a ratio of 1.95. The ratio values were then averaged to yield 1.7 + 0.35 and that value plotted in Fig 2.

| ~~Hybrid~~ Peptide | 10 µM uptake Well 1 | 10 µM uptake Well 2 | 10 µM uptake Well 3 | Average | Ratio of Averages | Ratio from independent run | Average of 2 runs |
|---|---|---|---|---|---|---|---|
| f-HN17-IR800 | 18285 | 20170 | 20642 | 19699±1247 | 1.45 | 1.95 | 1.7±0.25 (± PSD) |
| f-HN1-IR800 | 12233 | 39360 | 34125 | 28573±1439 | | | |

**Table S3.** The ratios below are plotted in Figure 2 from experiments performed as above reported in Table S2. The uncertainty shown is based upon population standard deviations from 2 – 3 independent experiments, except where indicated.

| ~~Hybrid~~ **Peptide** | **0.625 µM** | **1.25 µM** | **2.5 µM** | **5.0 µM** | **10 µM** |
|---|---|---|---|---|---|
| 4Iphf-HN17-IR800 | 0.226±0.011 | 0.502±0.031 | 1.207±0.348 | 2.417±0.199 | 4.497±0.963 # |
| f-HN17-IR800 | 0.101±0.001 | 0.291±0.043 | 0.491±0.130 | 1.083±0.030 | 1.700±0.247 # |
| f-HN1-IR800 | 0.107±0.023 | 0.258±0.061 | 0.343±0.029 | 0.693±0.075 | 1.000 # |
| HN1-IR800* | <LOD | 0.077±0.481 | 0.139±0.186 | 0.248±0.201 | 0.370±0.156 # |
| | | | | | |
| ~~Hybrid~~ **Peptide** | | **0.3 uM** | **1.0 uM** | **3 uM** | **10 uM** |
| 4Iph-HN1-IR800 | | 0.0214±0.021 | 0.069±0.040 | 0.231±0.036 | 0.563±0.032 |
| f-HNJ-IR800* | | 0.041±0.072 | 0.170±0.209 | 0.239±0.243 | 0.512±0.139 |
| HNJ-IR800* | | 0.101±0.088 | 0.058±0.100 | 0.139±0.095 | 0.316±0.129 |
| IR800-COOH | | 0.010±0.006 | 0.039±0.004 | 0.076±0.021 | 0.282±0.085 |

*Single standard experiment, average of 3 wells measured ± SD. # Single factor Anova $P < 0.05$ ($P = 0.011$, 0.008, and 0.012, respectively), for comparison of the 10 µM points, demonstrating significant differences from HN1-IR800. Adding f or 4Iph to HNJ-IR800 did not produce significant increases in cell uptake.
